# 2-(3-Cyano-4-{3-[1-(2-hy­droxy­eth­yl)-3,3-dimethyl-1,3-di­hydro­indol-2-yl­idene]prop-2-en­yl}-5,5-dimethyl-5*H*-furan-2-yl­idene)malono­nitrile

**DOI:** 10.1107/S1600536813033242

**Published:** 2013-12-14

**Authors:** Graeme J. Gainsford, M. Delower H. Bhuiyan, Andrew J. Kay

**Affiliations:** a Callaghan Innovation, PO Box 31-310, Lower Hutt, New Zealand

## Abstract

The title compound, C_25_H_24_N_4_O_2_, adopts a *cisoid* configuration and has twofold orientational disorder of the 2-hy­droxy­ethyl group. The mol­ecule is twisted from planarity so that the dihedral angle between the terminating indol-2-yl­idene and the furan-2-yl­idene moiety mean planes is 12.75 (7)°. Conformational disorder occurs at the indol-2-yl­idene N atom, which results in two orientations for the hy­droxy­ethyl group [occupancy ratio = 0.896 (2):0.104 (2)], and the hy­droxy O atom of the 2-hy­droxy­ethyl group is located over three sites [occupancy ratio = 0.548 (2):0.348 (2):0.104 (2)]. An intra­molecular C—H⋯O hydrogen bond involving the lowest occupancy hy­droxy O atom is observed. In the crystal, the mol­ecules pack in parallel dimeric sheets about centres of symmetry, utilizing O—H⋯N(cyano), C—H⋯N(cyano) and O—H⋯O hydrogen bonds, in two sets parallel to (02-1) and (021) planes.

## Related literature   

For general background to organic non-linear optical (NLO) materials and details of similar structures, see: Kay *et al.* (2004 ▶); Dalton *et al.* (1999 ▶); Harper *et al.* (1999 ▶); Kay *et al.* (2001*a* ▶,*b* ▶); Bhuiyan *et al.* (2011 ▶); Gainsford *et al.* (2011 ▶); Ma *et al.* (2002 ▶); Mao *et al.* (1998 ▶); Smith *et al.* (2010 ▶); Teshome *et al.* (2009 ▶). For the synthesis of the title compound, see: Bhuiyan *et al.* (2011 ▶). For the definition of bond-length alternation (BLA), see: Marder *et al.* (1993 ▶). For hydrogen-bond motifs, see: Bernstein *et al.* (1995 ▶). For details of the Cambridge Structural Database (CSD), see: Allen (2002 ▶).
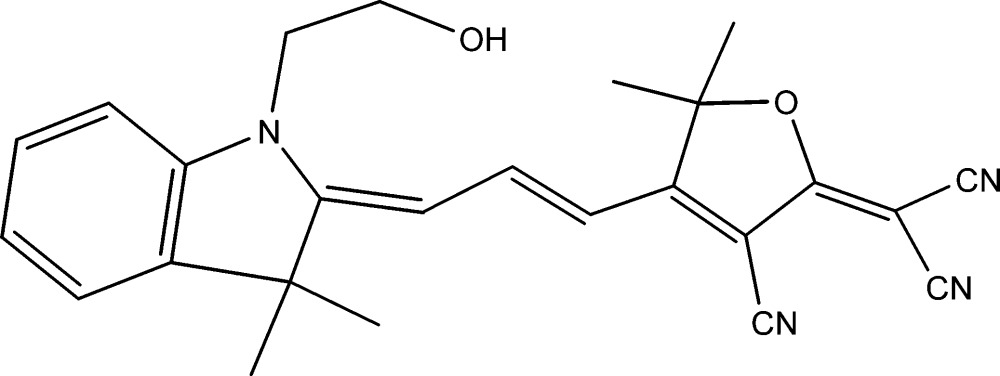


## Experimental   

### 

#### Crystal data  

C_25_H_24_N_4_O_2_
*M* *_r_* = 412.48
Monoclinic, 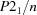

*a* = 9.4276 (4) Å
*b* = 21.5486 (9) Å
*c* = 11.1178 (5) Å
β = 103.916 (2)°
*V* = 2192.31 (16) Å^3^
*Z* = 4
Mo *K*α radiation
μ = 0.08 mm^−1^
*T* = 120 K
0.65 × 0.31 × 0.13 mm

#### Data collection  

Bruker–Nonius APEXII CCD area-detector diffractometer
Absorption correction: multi-scan (*SADABS*; Bruker, 2008 ▶) *T*_min_ = 0.629, *T*_max_ = 0.746
50145 measured reflections
6431 independent reflections
4754 reflections with *I* > 2σ(*I*)
*R*_int_ = 0.041

#### Refinement  

*R*[*F* ^2^ > 2σ(*F* ^2^)] = 0.056
*wR*(*F* ^2^) = 0.155
*S* = 1.03
6431 reflections
311 parameters
8 restraints
H atoms treated by a mixture of independent and constrained refinement
Δρ_max_ = 0.38 e Å^−3^
Δρ_min_ = −0.52 e Å^−3^

### 

Data collection: *APEX2* (Bruker, 2008 ▶); cell refinement: *SAINT* (Bruker, 2008 ▶); data reduction: *SAINT*; program(s) used to solve structure: *SHELXS97* (Sheldrick, 2008 ▶); program(s) used to refine structure: *SHELXL2012* (Sheldrick, 2008 ▶); molecular graphics: *ORTEP-3 for Windows* (Farrugia, 2012 ▶) and *Mercury* (Macrae *et al.*, 2006 ▶); software used to prepare material for publication: *SHELXL2012*, *PLATON* (Spek, 2009 ▶) and *Mercury* (Macrae *et al.*, 2006 ▶).

## Supplementary Material

Crystal structure: contains datablock(s) global, I. DOI: 10.1107/S1600536813033242/pk2507sup1.cif

Structure factors: contains datablock(s) I. DOI: 10.1107/S1600536813033242/pk2507Isup2.hkl

Supporting information file. DOI: 10.1107/S1600536813033242/pk2507Isup3.cml

Additional supporting information:  crystallographic information; 3D view; checkCIF report

## Figures and Tables

**Table 1:** Hydrogen-bond geometry (Å, °)

*D*—H⋯*A*	*D*—H	H⋯*A*	*D*⋯*A*	*D*—H⋯*A*
O2*A*1—H211⋯N3^i^	0.85 (3)	2.17 (4)	2.925 (3)	148 (6)
O2*A*2—H212⋯O2*A*1^ii^	0.84	2.27	2.939 (4)	137
C8—H8*B*⋯N1^iii^	0.98	2.62	3.501 (2)	150
C9—H9*C*⋯N3^iv^	0.98	2.60	3.539 (2)	160
C13—H13⋯O2*B*	0.95	2.57	3.299 (11)	134
C20—H20⋯N2^v^	0.95	2.65	3.442 (2)	141
C24*A*—H24*A*⋯N2^v^	0.99	2.59	3.555 (2)	166
C25*A*—H25*B*⋯N1^i^	0.99	2.55	3.348 (3)	137
C25*B*—H25*E*⋯N2^v^	0.99	2.45	3.420 (17)	167

Symmetry codes: (i) 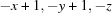; (ii) 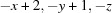; (iii) 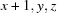; (iv) 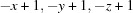; (v) 
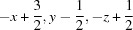
.

**Fig. 1.**
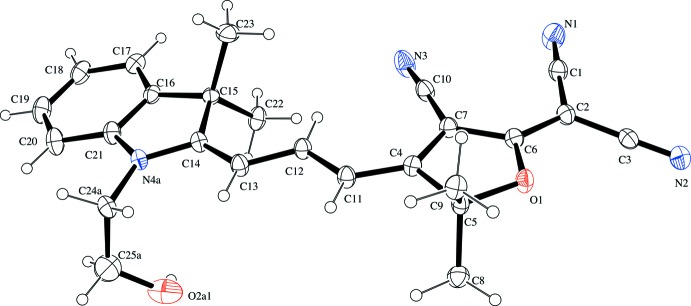
Molecular structure of the asymmetric unit (Farrugia, 2012); displacement ellipsoids are shown at the 30% probability level. Only the major conformation (A) of ring disorder involving atoms N4,C24,C25 & O2 are shown for clarity.

**Fig. 2.**
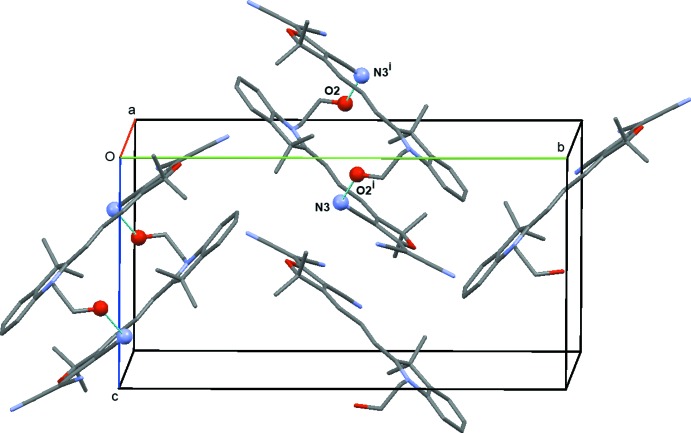
Packing diagram (Mercury; Macrae et al., 2006) of the unit cell; showing important hydrogen bonding as blue lines. Hydrogen atoms are omitted for clarity. The disordered atom O2A1 (see text) is shown labeled as O2; symmetry operation: (i) 1 - x,1 - y,-z.

## supplementary crystallographic information

### 1. Comment

Organic nonlinear optical (NLO) chromophores containing donor (D) and acceptor (A) units have been widely reported in the literature as the enabling materials for a range of photonic devices (Dalton *et al.*, 1999; Mao *et al.*, 1998; Harper *et al.*, 1999; Ma *et al.*, 2002). We have previously reported a synthetic methodology (Kay *et al.*, 2001*a*; Kay *et al.*, 2001*b*, Teshome *et al.*, 2009; Kay *et al.*, 2004; Smith *et al.*, 2010) that allows entry to a number of high figure-of-merit NLO chromophores with aromatisable donors (*e.g.* 1,4-dihydropyridinylidene, 1,4-dihydroquinolinylidene), and containing the powerful acceptor 4,5,5-trimethyl-3-cyano-2(5*H*)-furanylidenepropane dinitrile (TCF). While this approach allowed for ease of synthesis and for a controlled increase in the extent of conjugation in the molecules, the resultant 'parent' merocyanines are prone to significant amounts of aggregation (Teshome *et al.*, 2009). As a continuation of this work, we have further developed our synthetic methodology, and extend the series to include chromophores with a non-aromatisable indoline donor group. Here we have synthesized a new NLO chromophore containing an indoline donor, an acceptor based on the well known moiety (2-(3-cyano-4,5,5-trimethyl-5*H*-furan-2-ylidene)-malononitrile), hereafter CTF, and a conjugated chain of three carbon atoms between the donor and acceptor. The chromophore also contains an hydroxyethyl substituent on the donor nitrogen atom which will allow for covalent attachment of the molecule to a polymer backbone, if needed, in the future.

The asymmetric unit (Figure 1) illustrates the *cisoid* configuration as compared with the *transoid* configurations found in the closely related 2-(3-cyano-4-{5-[1-(2-hydroxy-ethyl)-3,3-dimethyl-1,3-dihydro- indol-2-ylidene]-penta-1,3-dienyl}-5,5-dimethyl-5*H*-furan-2-ylidene) -malononitrile (hereafter TMIPNS, Bhuiyan *et al.*, 2011) and 2-(3-cyano-4-{7-[1-(2-hydroxyethyl)-3,3-dimethylindolin-2-ylidene]hepta-1,3,5-trienyl}-5,5-dimethyl-2,5-dihydrofuran-2-ylidene)malononitrile (Gainsford *et al.*, 2011). The bond length alternation value, BLA (Marder *et al.*, 1993) is 0.011 Å compared with 0.024 Å in related compound TMIPNS. The molecule is twisted from planarity so that the dihedral angle between the terminating indol-2-ylidene and furan-2-ylidene moiety planes is 12.75 (7)%. This twist is less that in TMIPNS (twisted ~19°) but both molecules bend into a similar shape.

The indol-2-ylidene moieties (major conformation) have planar 5- and 6-membered rings that subtend small angles, here 1.75 (8)°, compared with 1.95 (11)° in TMIPNS. The planes through the planar entities (indol-2-ylidene ring, polyene atoms (C11–C13) & five membered ring (C4–C7,O1) are twisted progressively by 9.13 (17) & 5.72 (17)°. These values compare with 8.66 (19) & 9.2 (2)° in TMIPNS, meaning the polyene chain is slightly more coplanar here with the 5-membered furan-2-ylidene ring.

The molecules pack in parallel dimeric sheets about centres of symmetry utilizing an *O*–*H*···*N*(cyano) hydrogen bond (Figure 2) in two sets parallel to (021) and (021) planes with principal motif *R*^2^_2_(26) (Bernstein *et al.*, 1995). Other weaker interactions *C*–*H*···*N*(cyano), and also involving the disordered hydroxy atoms (*O*–*H*···*O*) are observed (Table 1).

### 2. Experimental

We have synthesized the title compound by following the procedure in Bhuiyan *et al.* (2011). Single crystals were grown by slow ether diffusion into a dichloromethane solution of the compound.

### 3. Refinement

A total of 4 reflections within 2θ 55° were omitted as being partially screened by the backstop. The 5-membered C14—C16,N4 ring was disordered over two sites [0.896 (2):0.104 (2)], giving two major orientations for the 2-hydroxy-ethyl substituents. The oxygen atoms of the hydroxy group on the major conformation (labelled A) were also disordered over two sites; a restraint was applied (SUMP) to ensure total occupancy of the hydroxy OH atoms (3 sites) was unity. To ensure reasonable connectivity, C25A–H and C25A–O bond lengths were restrained to be the same, and all hydroxy O2–H bonds were fixed at 0.84 Å. The hydroxy H atoms were located and refined with *U*_iso_ = 1.5*U*_eq_(O). Terminal atoms on the 2-hydroxy ethyl substituents except O2A1 were refined with isotropic linked thermal parameters. Hydrogen atoms bound to carbon were constrained to their expected geometries (C—H 0.98, 0.99 Å): all methyl and tertiary H atoms were refined with *U*_iso_ 1.5 or 1.2 times respectively that of the *U*_eq_ of their parent atom.

### Crystal data

**Table:**

C_25_H_24_N_4_O_2_	*F*(000) = 872
*M**_r_* = 412.48	*D*_x_ = 1.250 Mg m^−^^3^
Monoclinic, *P*2_1_/*n*	Mo *K*α radiation, λ = 0.71073 Å
Hall symbol: -P 2yn	Cell parameters from 9996 reflections
*a* = 9.4276 (4) Å	θ = 2.4–30.0°
*b* = 21.5486 (9) Å	µ = 0.08 mm^−^^1^
*c* = 11.1178 (5) Å	*T* = 120 K
β = 103.916 (2)°	Block, blue
*V* = 2192.31 (16) Å^3^	0.65 × 0.31 × 0.13 mm
*Z* = 4	

### Data collection

**Table:**

Bruker–Nonius APEXII CCD area-detector diffractometer	6431 independent reflections
Radiation source: fine-focus sealed tube	4754 reflections with *I* > 2σ(*I*)
Graphite monochromator	*R*_int_ = 0.041
Detector resolution: 8.333 pixels mm^-1^	θ_max_ = 30.2°, θ_min_ = 2.7°
φ and ω scans	*h* = −13→12
Absorption correction: multi-scan (*SADABS*; Bruker, 2008)	*k* = −30→30
*T*_min_ = 0.629, *T*_max_ = 0.746	*l* = −15→15
50145 measured reflections	

### Refinement

**Table:**

Refinement on *F*^2^	Primary atom site location: structure-invariant direct methods
Least-squares matrix: full	Secondary atom site location: difference Fourier map
*R*[*F*^2^ > 2σ(*F*^2^)] = 0.056	Hydrogen site location: inferred from neighbouring sites
*wR*(*F*^2^) = 0.155	H atoms treated by a mixture of independent and constrained refinement
*S* = 1.03	*w* = 1/[σ^2^(*F*_o_^2^) + (0.0688*P*)^2^ + 1.0008*P*] where *P* = (*F*_o_^2^ + 2*F*_c_^2^)/3
6431 reflections	(Δ/σ)_max_ < 0.001
311 parameters	Δρ_max_ = 0.38 e Å^−^^3^
8 restraints	Δρ_min_ = −0.52 e Å^−^^3^

### Special details

**Table:**

Geometry. All e.s.d.'s (except the e.s.d. in the dihedral angle between two l.s. planes) are estimated using the full covariance matrix. The cell e.s.d.'s are taken into account individually in the estimation of e.s.d.'s in distances, angles and torsion angles; correlations between e.s.d.'s in cell parameters are only used when they are defined by crystal symmetry. An approximate (isotropic) treatment of cell e.s.d.'s is used for estimating e.s.d.'s involving l.s. planes.
Refinement. Refinement of *F*^2^ against ALL reflections. The weighted *R*-factor *wR* and goodness of fit *S* are based on *F*^2^, conventional *R*-factors *R* are based on *F*, with *F* set to zero for negative *F*^2^. The threshold expression of *F*^2^ > σ(*F*^2^) is used only for calculating *R*-factors(gt) *etc*. and is not relevant to the choice of reflections for refinement. *R*-factors based on *F*^2^ are statistically about twice as large as those based on *F*, and *R*- factors based on ALL data will be even larger.

### Fractional atomic coordinates and isotropic or equivalent isotropic displacement parameters (Å^2^)

**Table:**

	*x*	*y*	*z*	*U*_iso_*/*U*_eq_	Occ. (<1)
O1	0.63766 (11)	0.63737 (5)	0.49304 (11)	0.0335 (2)	
N1	0.14137 (16)	0.57222 (8)	0.39517 (18)	0.0514 (4)	
N2	0.36701 (16)	0.73490 (7)	0.56984 (15)	0.0421 (3)	
N3	0.34340 (16)	0.48266 (8)	0.24877 (17)	0.0518 (4)	
C1	0.25102 (16)	0.59701 (7)	0.42748 (15)	0.0344 (3)	
C2	0.38498 (15)	0.62889 (7)	0.46900 (14)	0.0302 (3)	
C3	0.37879 (16)	0.68746 (7)	0.52649 (14)	0.0319 (3)	
C4	0.68760 (15)	0.55350 (6)	0.37397 (13)	0.0273 (3)	
C5	0.75963 (14)	0.60753 (7)	0.45254 (14)	0.0281 (3)	
C6	0.51422 (15)	0.60667 (7)	0.44612 (13)	0.0282 (3)	
C7	0.53982 (15)	0.55468 (7)	0.37537 (13)	0.0283 (3)	
C8	0.81902 (17)	0.65591 (7)	0.37893 (16)	0.0361 (3)	
H8A	0.7422	0.6683	0.3067	0.054*	
H8B	0.9018	0.6385	0.3511	0.054*	
H8C	0.8516	0.6923	0.4313	0.054*	
C9	0.87158 (16)	0.58711 (7)	0.56768 (14)	0.0335 (3)	
H9A	0.9087	0.6235	0.6184	0.050*	
H9B	0.9527	0.5661	0.5435	0.050*	
H9C	0.8258	0.5585	0.6156	0.050*	
C10	0.42912 (16)	0.51515 (8)	0.30741 (15)	0.0342 (3)	
C11	0.76702 (15)	0.51590 (7)	0.31209 (14)	0.0300 (3)	
H11	0.8687	0.5239	0.3253	0.036*	
C12	0.70918 (15)	0.46773 (7)	0.23262 (13)	0.0288 (3)	
H12	0.6101	0.4564	0.2253	0.035*	
C13	0.78859 (16)	0.43509 (7)	0.16310 (14)	0.0324 (3)	
H13	0.8894	0.4447	0.1757	0.039*	
C14	0.73222 (15)	0.38957 (7)	0.07677 (13)	0.0289 (3)	
C15	0.57736 (15)	0.36373 (6)	0.03954 (13)	0.0260 (3)	
C16	0.58984 (16)	0.31748 (6)	−0.05962 (13)	0.0280 (3)	
C17	0.48672 (18)	0.27866 (7)	−0.13049 (15)	0.0358 (3)	
H17	0.3902	0.2770	−0.1189	0.043*	
C18	0.5276 (2)	0.24182 (8)	−0.21974 (16)	0.0418 (4)	
H18	0.4575	0.2153	−0.2705	0.050*	
C19	0.6681 (2)	0.24341 (9)	−0.23514 (16)	0.0451 (4)	
H19	0.6932	0.2181	−0.2969	0.054*	
C20	0.7737 (2)	0.28110 (9)	−0.16259 (16)	0.0445 (4)	
H20	0.8713	0.2817	−0.1717	0.053*	
C21	0.72999 (17)	0.31800 (8)	−0.07588 (14)	0.0344 (3)	
C22	0.46596 (17)	0.41387 (8)	−0.01844 (16)	0.0374 (3)	
H22A	0.5009	0.4360	−0.0827	0.056*	
H22B	0.4543	0.4432	0.0458	0.056*	
H22C	0.3717	0.3943	−0.0554	0.056*	
C23	0.5323 (2)	0.33128 (8)	0.14723 (16)	0.0458 (4)	
H23A	0.4404	0.3089	0.1158	0.069*	
H23B	0.5190	0.3623	0.2080	0.069*	
H23C	0.6087	0.3019	0.1868	0.069*	
N4A	0.81429 (15)	0.35927 (7)	0.01028 (14)	0.0290 (3)	0.896 (2)
C24A	0.96803 (18)	0.37120 (9)	0.01489 (17)	0.0356 (4)	0.896 (2)
H24A	1.0180	0.3315	0.0076	0.043*	0.896 (2)
H24B	1.0155	0.3901	0.0957	0.043*	0.896 (2)
C25A	0.9838 (3)	0.41401 (13)	−0.0880 (3)	0.0596 (6)*	0.896 (2)
H25A	1.0890	0.4183	−0.0856	0.072*	0.548 (2)
H25B	0.9358	0.3946	−0.1682	0.072*	0.548 (2)
O2A1	0.9263 (3)	0.47186 (17)	−0.0833 (3)	0.0675 (9)	0.548 (2)
H211	0.844 (4)	0.469 (3)	−0.136 (5)	0.101*	0.548 (2)
O2A2	1.1107 (5)	0.4305 (2)	−0.0912 (4)	0.0596 (6)*	0.348 (2)
H212	1.139 (9)	0.447 (4)	−0.021 (4)	0.089*	0.348 (2)
H25C	0.959 (8)	0.386 (3)	−0.161 (5)	0.089*	0.348 (2)
H25D	0.905 (8)	0.444 (4)	−0.120 (10)	0.089*	0.348 (2)
N4B	0.7997 (16)	0.3841 (8)	−0.0305 (15)	0.0384 (19)*	0.104 (2)
C24B	0.9155 (19)	0.4140 (8)	−0.0540 (16)	0.0384 (19)*	0.104 (2)
H24C	0.9153	0.4570	−0.0226	0.046*	0.104 (2)
H24D	0.9011	0.4167	−0.1451	0.046*	0.104 (2)
C25B	1.0719 (17)	0.3854 (8)	0.0016 (12)	0.0384 (19)*	0.104 (2)
H25E	1.0732	0.3417	−0.0258	0.046*	0.104 (2)
H25F	1.1455	0.4086	−0.0307	0.046*	0.104 (2)
O2B	1.1083 (12)	0.3875 (5)	0.1268 (10)	0.0384 (19)*	0.104 (2)
H2B	1.0676	0.4182	0.1509	0.058*	0.104 (2)

### Atomic displacement parameters (Å^2^)

**Table:**

	*U*^11^	*U*^22^	*U*^33^	*U*^12^	*U*^13^	*U*^23^
O1	0.0254 (5)	0.0339 (5)	0.0413 (6)	0.0018 (4)	0.0080 (4)	−0.0115 (5)
N1	0.0296 (7)	0.0499 (9)	0.0738 (11)	0.0026 (6)	0.0107 (7)	−0.0096 (8)
N2	0.0428 (8)	0.0404 (8)	0.0472 (8)	0.0028 (6)	0.0186 (6)	−0.0045 (6)
N3	0.0327 (7)	0.0672 (11)	0.0592 (10)	−0.0116 (7)	0.0184 (7)	−0.0276 (8)
C1	0.0285 (7)	0.0352 (8)	0.0401 (8)	0.0078 (6)	0.0094 (6)	−0.0005 (6)
C2	0.0278 (7)	0.0325 (7)	0.0312 (7)	0.0042 (5)	0.0086 (5)	−0.0001 (6)
C3	0.0293 (7)	0.0372 (8)	0.0315 (7)	0.0037 (6)	0.0119 (6)	0.0022 (6)
C4	0.0252 (6)	0.0279 (6)	0.0273 (7)	0.0021 (5)	0.0032 (5)	−0.0018 (5)
C5	0.0221 (6)	0.0286 (7)	0.0334 (7)	0.0027 (5)	0.0063 (5)	−0.0055 (5)
C6	0.0261 (6)	0.0304 (7)	0.0276 (7)	0.0025 (5)	0.0053 (5)	0.0002 (5)
C7	0.0249 (6)	0.0304 (7)	0.0289 (7)	0.0016 (5)	0.0051 (5)	−0.0024 (5)
C8	0.0343 (7)	0.0324 (7)	0.0415 (9)	0.0020 (6)	0.0090 (6)	0.0018 (6)
C9	0.0301 (7)	0.0350 (8)	0.0326 (7)	0.0008 (6)	0.0020 (6)	−0.0045 (6)
C10	0.0265 (7)	0.0414 (8)	0.0368 (8)	−0.0010 (6)	0.0114 (6)	−0.0074 (7)
C11	0.0236 (6)	0.0328 (7)	0.0325 (7)	0.0026 (5)	0.0046 (5)	−0.0055 (6)
C12	0.0254 (6)	0.0308 (7)	0.0294 (7)	0.0027 (5)	0.0050 (5)	−0.0037 (5)
C13	0.0250 (6)	0.0367 (8)	0.0357 (8)	0.0010 (5)	0.0077 (5)	−0.0079 (6)
C14	0.0273 (6)	0.0324 (7)	0.0276 (7)	0.0044 (5)	0.0077 (5)	−0.0021 (5)
C15	0.0285 (6)	0.0254 (6)	0.0245 (6)	0.0022 (5)	0.0072 (5)	0.0017 (5)
C16	0.0342 (7)	0.0250 (6)	0.0238 (6)	0.0051 (5)	0.0050 (5)	0.0021 (5)
C17	0.0409 (8)	0.0308 (7)	0.0334 (8)	0.0008 (6)	0.0046 (6)	−0.0004 (6)
C18	0.0541 (10)	0.0329 (8)	0.0327 (8)	0.0037 (7)	−0.0003 (7)	−0.0061 (6)
C19	0.0556 (10)	0.0445 (9)	0.0315 (8)	0.0129 (8)	0.0034 (7)	−0.0115 (7)
C20	0.0431 (9)	0.0535 (10)	0.0363 (9)	0.0114 (8)	0.0088 (7)	−0.0128 (8)
C21	0.0357 (7)	0.0380 (8)	0.0284 (7)	0.0066 (6)	0.0059 (6)	−0.0054 (6)
C22	0.0326 (7)	0.0357 (8)	0.0395 (8)	0.0093 (6)	0.0003 (6)	−0.0047 (7)
C23	0.0701 (12)	0.0383 (9)	0.0351 (9)	−0.0105 (8)	0.0246 (8)	−0.0007 (7)
N4A	0.0284 (7)	0.0327 (7)	0.0270 (7)	0.0038 (5)	0.0088 (5)	−0.0032 (6)
C24A	0.0288 (8)	0.0422 (9)	0.0379 (9)	0.0061 (7)	0.0121 (7)	−0.0006 (7)
O2A1	0.0470 (15)	0.081 (2)	0.069 (2)	−0.0066 (14)	0.0018 (13)	0.0281 (17)

### Geometric parameters (Å, º)

**Table:**

O1—C6	1.3312 (17)	C16—C17	1.377 (2)
O1—C5	1.4790 (16)	C17—C18	1.395 (2)
N1—C1	1.142 (2)	C17—H17	0.9500
N2—C3	1.147 (2)	C18—C19	1.377 (3)
N3—C10	1.147 (2)	C18—H18	0.9500
C1—C2	1.414 (2)	C19—C20	1.384 (3)
C2—C6	1.3883 (19)	C19—H19	0.9500
C2—C3	1.422 (2)	C20—C21	1.386 (2)
C4—C11	1.3922 (19)	C20—H20	0.9500
C4—C7	1.3972 (19)	C21—N4A	1.405 (2)
C4—C5	1.5148 (19)	C21—N4B	1.599 (16)
C5—C8	1.513 (2)	C22—H22A	0.9800
C5—C9	1.515 (2)	C22—H22B	0.9800
C6—C7	1.423 (2)	C22—H22C	0.9800
C7—C10	1.416 (2)	C23—H23A	0.9800
C8—H8A	0.9800	C23—H23B	0.9800
C8—H8B	0.9800	C23—H23C	0.9800
C8—H8C	0.9800	N4A—C24A	1.461 (2)
C9—H9A	0.9800	C24A—C25A	1.504 (3)
C9—H9B	0.9800	C24A—H24A	0.9900
C9—H9C	0.9800	C24A—H24B	0.9900
C11—C12	1.387 (2)	C25A—O2A1	1.365 (4)
C11—H11	0.9500	C25A—H25A	0.9900
C12—C13	1.389 (2)	C25A—H25B	0.9900
C12—H12	0.9500	O2A1—H211	0.85 (3)
C13—C14	1.386 (2)	O2A2—H212	0.84 (3)
C13—H13	0.9500	N4B—C24B	1.35 (2)
C14—N4A	1.3588 (19)	C24B—C25B	1.58 (2)
C14—N4B	1.485 (16)	C24B—H24C	0.9900
C14—C15	1.524 (2)	C24B—H24D	0.9900
C15—C16	1.5116 (19)	C25B—O2B	1.352 (13)
C15—C23	1.532 (2)	C25B—H25E	0.9900
C15—C22	1.536 (2)	C25B—H25F	0.9900
C16—C21	1.376 (2)	O2B—H2B	0.8400
			
C6—O1—C5	109.57 (11)	C18—C17—H17	120.8
N1—C1—C2	178.50 (18)	C19—C18—C17	120.84 (16)
C6—C2—C1	121.79 (14)	C19—C18—H18	119.6
C6—C2—C3	121.41 (14)	C17—C18—H18	119.6
C1—C2—C3	116.61 (13)	C18—C19—C20	121.40 (16)
N2—C3—C2	176.76 (17)	C18—C19—H19	119.3
C11—C4—C7	132.24 (13)	C20—C19—H19	119.3
C11—C4—C5	120.83 (12)	C19—C20—C21	116.72 (16)
C7—C4—C5	106.83 (12)	C19—C20—H20	121.6
O1—C5—C8	106.32 (11)	C21—C20—H20	121.6
O1—C5—C4	103.66 (10)	C16—C21—C20	122.82 (15)
C8—C5—C4	112.99 (13)	C16—C21—N4A	108.60 (13)
O1—C5—C9	107.67 (12)	C20—C21—N4A	128.55 (15)
C8—C5—C9	112.56 (12)	C16—C21—N4B	107.5 (6)
C4—C5—C9	112.85 (12)	C20—C21—N4B	124.2 (6)
O1—C6—C2	118.67 (13)	C15—C22—H22A	109.5
O1—C6—C7	111.07 (12)	C15—C22—H22B	109.5
C2—C6—C7	130.24 (14)	H22A—C22—H22B	109.5
C4—C7—C10	126.29 (13)	C15—C22—H22C	109.5
C4—C7—C6	108.83 (12)	H22A—C22—H22C	109.5
C10—C7—C6	124.56 (13)	H22B—C22—H22C	109.5
C5—C8—H8A	109.5	C15—C23—H23A	109.5
C5—C8—H8B	109.5	C15—C23—H23B	109.5
H8A—C8—H8B	109.5	H23A—C23—H23B	109.5
C5—C8—H8C	109.5	C15—C23—H23C	109.5
H8A—C8—H8C	109.5	H23A—C23—H23C	109.5
H8B—C8—H8C	109.5	H23B—C23—H23C	109.5
C5—C9—H9A	109.5	C14—N4A—C21	111.86 (13)
C5—C9—H9B	109.5	C14—N4A—C24A	125.91 (14)
H9A—C9—H9B	109.5	C21—N4A—C24A	121.93 (13)
C5—C9—H9C	109.5	N4A—C24A—C25A	111.10 (17)
H9A—C9—H9C	109.5	N4A—C24A—H24A	109.4
H9B—C9—H9C	109.5	C25A—C24A—H24A	109.4
N3—C10—C7	176.92 (17)	N4A—C24A—H24B	109.4
C12—C11—C4	125.07 (13)	C25A—C24A—H24B	109.4
C12—C11—H11	117.5	H24A—C24A—H24B	108.0
C4—C11—H11	117.5	O2A1—C25A—C24A	114.7 (2)
C11—C12—C13	123.45 (13)	O2A1—C25A—H25A	108.6
C11—C12—H12	118.3	C24A—C25A—H25A	108.6
C13—C12—H12	118.3	O2A1—C25A—H25B	108.6
C14—C13—C12	125.12 (13)	C24A—C25A—H25B	108.6
C14—C13—H13	117.4	H25A—C25A—H25B	107.6
C12—C13—H13	117.4	C25A—O2A1—H211	103 (5)
N4A—C14—C13	122.90 (13)	C24B—N4B—C14	130.0 (13)
C13—C14—N4B	116.4 (6)	C24B—N4B—C21	131.0 (13)
N4A—C14—C15	108.08 (12)	C14—N4B—C21	95.8 (9)
C13—C14—C15	129.02 (13)	N4B—C24B—C25B	117.4 (15)
N4B—C14—C15	108.7 (6)	N4B—C24B—H24C	107.9
C16—C15—C14	101.62 (11)	C25B—C24B—H24C	107.9
C16—C15—C23	110.73 (12)	N4B—C24B—H24D	107.9
C14—C15—C23	112.45 (13)	C25B—C24B—H24D	107.9
C16—C15—C22	108.84 (12)	H24C—C24B—H24D	107.2
C14—C15—C22	111.76 (12)	O2B—C25B—C24B	111.8 (13)
C23—C15—C22	111.03 (13)	O2B—C25B—H25E	109.3
C21—C16—C17	119.85 (14)	C24B—C25B—H25E	109.3
C21—C16—C15	109.64 (13)	O2B—C25B—H25F	109.3
C17—C16—C15	130.50 (14)	C24B—C25B—H25F	109.3
C16—C17—C18	118.35 (16)	H25E—C25B—H25F	107.9
C16—C17—H17	120.8	C25B—O2B—H2B	109.5
			
C6—O1—C5—C8	119.01 (13)	C21—C16—C17—C18	−1.6 (2)
C6—O1—C5—C4	−0.33 (15)	C15—C16—C17—C18	177.65 (14)
C6—O1—C5—C9	−120.13 (13)	C16—C17—C18—C19	1.1 (2)
C11—C4—C5—O1	175.98 (13)	C17—C18—C19—C20	0.4 (3)
C7—C4—C5—O1	−0.88 (15)	C18—C19—C20—C21	−1.4 (3)
C11—C4—C5—C8	61.32 (18)	C17—C16—C21—C20	0.6 (2)
C7—C4—C5—C8	−115.54 (13)	C15—C16—C21—C20	−178.78 (15)
C11—C4—C5—C9	−67.82 (18)	C17—C16—C21—N4A	−177.63 (14)
C7—C4—C5—C9	115.32 (13)	C15—C16—C21—N4A	2.97 (17)
C5—O1—C6—C2	−177.33 (13)	C17—C16—C21—N4B	155.4 (6)
C5—O1—C6—C7	1.43 (16)	C15—C16—C21—N4B	−24.0 (7)
C1—C2—C6—O1	−176.75 (14)	C19—C20—C21—C16	0.9 (3)
C3—C2—C6—O1	8.5 (2)	C19—C20—C21—N4A	178.75 (17)
C1—C2—C6—C7	4.8 (3)	C19—C20—C21—N4B	−149.7 (7)
C3—C2—C6—C7	−169.99 (15)	C13—C14—N4A—C21	−176.54 (15)
C11—C4—C7—C10	−0.9 (3)	N4B—C14—N4A—C21	−91.8 (13)
C5—C4—C7—C10	175.45 (15)	C15—C14—N4A—C21	4.19 (18)
C11—C4—C7—C6	−174.63 (16)	C13—C14—N4A—C24A	−2.7 (3)
C5—C4—C7—C6	1.72 (16)	N4B—C14—N4A—C24A	82.0 (13)
O1—C6—C7—C4	−2.04 (17)	C15—C14—N4A—C24A	177.99 (15)
C2—C6—C7—C4	176.53 (15)	C16—C21—N4A—C14	−4.59 (19)
O1—C6—C7—C10	−175.90 (14)	C20—C21—N4A—C14	177.29 (17)
C2—C6—C7—C10	2.7 (3)	N4B—C21—N4A—C14	87.2 (13)
C7—C4—C11—C12	−0.2 (3)	C16—C21—N4A—C24A	−178.68 (15)
C5—C4—C11—C12	−176.12 (14)	C20—C21—N4A—C24A	3.2 (3)
C4—C11—C12—C13	173.48 (15)	N4B—C21—N4A—C24A	−86.9 (13)
C11—C12—C13—C14	−175.49 (15)	C14—N4A—C24A—C25A	−96.2 (2)
C12—C13—C14—N4A	179.46 (16)	C21—N4A—C24A—C25A	77.1 (2)
C12—C13—C14—N4B	148.2 (7)	N4A—C24A—C25A—O2A1	62.7 (3)
C12—C13—C14—C15	−1.4 (3)	N4A—C14—N4B—C24B	−106 (2)
N4A—C14—C15—C16	−2.18 (15)	C13—C14—N4B—C24B	5 (2)
C13—C14—C15—C16	178.62 (15)	C15—C14—N4B—C24B	160.6 (16)
N4B—C14—C15—C16	27.1 (7)	N4A—C14—N4B—C21	55.0 (10)
N4A—C14—C15—C23	116.24 (15)	C13—C14—N4B—C21	166.0 (4)
C13—C14—C15—C23	−63.0 (2)	C15—C14—N4B—C21	−38.5 (9)
N4B—C14—C15—C23	145.5 (7)	C16—C21—N4B—C24B	−161.1 (16)
N4A—C14—C15—C22	−118.10 (14)	C20—C21—N4B—C24B	−7 (2)
C13—C14—C15—C22	62.7 (2)	N4A—C21—N4B—C24B	102 (2)
N4B—C14—C15—C22	−88.8 (7)	C16—C21—N4B—C14	38.2 (9)
C14—C15—C16—C21	−0.53 (15)	C20—C21—N4B—C14	−167.5 (3)
C23—C15—C16—C21	−120.18 (15)	N4A—C21—N4B—C14	−58.5 (10)
C22—C15—C16—C21	117.51 (14)	C14—N4B—C24B—C25B	87 (2)
C14—C15—C16—C17	−179.84 (15)	C21—N4B—C24B—C25B	−68 (2)
C23—C15—C16—C17	60.5 (2)	N4B—C24B—C25B—O2B	−65.8 (19)
C22—C15—C16—C17	−61.81 (19)		

### Hydrogen-bond geometry (Å, º)

**Table:**

*D*—H···*A*	*D*—H	H···*A*	*D*···*A*	*D*—H···*A*
O2*A*1—H211···N3^i^	0.85 (3)	2.17 (4)	2.925 (3)	148 (6)
O2*A*2—H212···O2*A*1^ii^	0.84	2.27	2.939 (4)	137
C8—H8*B*···N1^iii^	0.98	2.62	3.501 (2)	150
C9—H9*C*···N3^iv^	0.98	2.60	3.539 (2)	160
C13—H13···O2*B*	0.95	2.57	3.299 (11)	134
C20—H20···N2^v^	0.95	2.65	3.442 (2)	141
C24*A*—H24*A*···N2^v^	0.99	2.59	3.555 (2)	166
C25*A*—H25*B*···N1^i^	0.99	2.55	3.348 (3)	137
C25*B*—H25*E*···N2^v^	0.99	2.45	3.420 (17)	167

Symmetry codes: (i) −*x*+1, −*y*+1, −*z*; (ii) −*x*+2, −*y*+1, −*z*; (iii) *x*+1, *y*, *z*; (iv) −*x*+1, −*y*+1, −*z*+1; (v) −*x*+3/2, *y*−1/2, −*z*+1/2.
